# Mechanisms of ciliogenesis suppression in dividing cells

**DOI:** 10.1007/s00018-016-2369-9

**Published:** 2016-09-26

**Authors:** Hidemasa Goto, Hironori Inaba, Masaki Inagaki

**Affiliations:** 1 Division of Biochemistry, Aichi Cancer Center Research Institute, Nagoya, 464-8681 Japan; 2 Department of Cellular Oncology, Graduate School of Medicine, Nagoya University, Nagoya, 466-8550 Japan; 3 Department of Physiology, Mie University School of Medicine, Tsu, Mie Japan

**Keywords:** Primary cilia, Cell cycle, Aurora-A, Cancer, Ciliopathy

## Abstract

The primary cilium is a non-motile and microtubule-enriched protrusion ensheathed by plasma membrane. Primary cilia function as mechano/chemosensors and signaling hubs and their disorders predispose to a wide spectrum of human diseases. Most types of cells assemble their primary cilia in response to cellular quiescence, whereas they start to retract the primary cilia upon cell-cycle reentry. The retardation of ciliary resorption process has been shown to delay cell-cycle progression to the S or M phase after cell-cycle reentry. Apart from this conventional concept of ciliary disassembly linked to cell-cycle reentry, recent studies have led to a novel concept, suggesting that cells can suppress primary cilia assembly during cell proliferation. Accumulating evidence has also demonstrated the importance of Aurora-A (a protein originally identified as one of mitotic kinases) not only in ciliary resorption after cell-cycle reentry but also in the suppression of ciliogenesis in proliferating cells, whereas Aurora-A activators are clearly distinct in both phenomena. Here, we summarize the current knowledge of how cycling cells suppress ciliogenesis and compare it with mechanisms underlying ciliary resorption after cell-cycle reentry. We also discuss a reciprocal relationship between primary cilia and cell proliferation.

## Introduction

A primary cilium, a solitary projection from the apical cell surface, exists in the majority of cells in the human body. The primary cilium functions not only as a sensory organelle to detect extracellular cues, such as mechanical flow, but also as an antenna to transduce extracellular signals, such as growth factors, hormones, and developmental morphogens, into the cell [1–5]. Defects in ciliary structure and function are associated with a broad spectrum of diseases (termed ciliopathies), such as polydactyly, cranio-facial abnormalities, brain malformation, congenital heart diseases, situs inversus (defects of left–right patterning), obesity, diabetes, and polycystic kidney disease (PKD) [6–10].

**Fig. 1:**
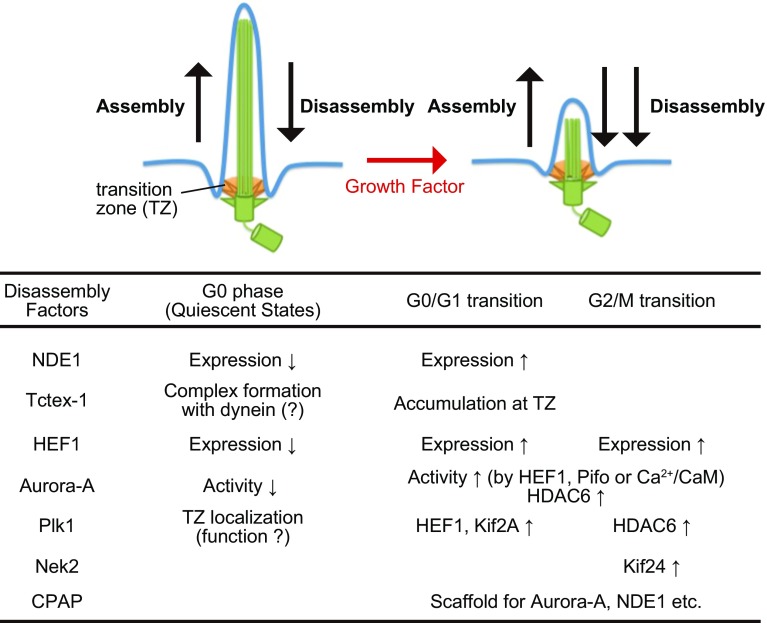
Summary of representative deciliation factors after cell-cycle reentry. NDE1 and Tctex-1 negatively control ciliary length during the G0 phase when it becomes constant. Their deciliation activity is elevated at the G0/G1 transition. Other factors are unlikely to participate in the maintenance of primary cilia during quiescent state

The primary cilium consists of a basal body, an axoneme, and a transition zone [13–15]; also see Fig. 1. The basal body originates from a mother centriole on a centrosome, whereas the axoneme is a microtubule-based structure sheathed by the ciliary membrane, a lipid bilayer distinct in composition from the plasma membrane [11, 12]. The transition zone represents a boundary architecture between the above two structures. Accumulating evidence has suggested that ciliary assembly requires different types of proteins, including membrane vesicle trafficking proteins, such as a small GTPase Rab8, its specific GTP exchange factor Rabin 8, and a complex of proteins encoded by genes mutated in Bardet–Biedl Syndrome; proteins localized at appendages on mother centrioles, such as ODF2/hCenexin, CEP164, CEP89/CCDC123, CEP83, SCLT1, and FBF1/Albatross; ciliary anterograde transport protein complex, such as Kinesin-2 family protein and IFT complex B; and proteins implicated in the ciliopathy Meckel–Gruber syndrome, such as MKS1 and MKS3 [14–20]. Recent studies have also identified several negative regulators, including capping proteins at distal ends of mother centrioles, such as CP110; constituent proteins of the dynein complex, such as NDE1 and Tctex-1; microtubule depolymerizing kinesins, including KIF2A, KIF19A, and KIF24; mitotic kinases, including Aurora-A and PLK1; Aurora-A-associated proteins, such as HEF1, calcium-calmodulin (Ca^2+^/CaM), Pitchfork (Pifo), and trichoplein; and a tubulin deacetylase HDAC6 [15–17, 21–23]. OFD1 (Orofaciodigital syndrome 1) appears to regulate ciliogenesis both positively and negatively [24–26].

Typically, primary cilia start to form during the quiescent state (the G0 phase): we use the G0 phase to distinguish proliferative G1 phase [followed by the S phase (DNA replication)], although it is still a matter of debate as to whether the G0 phase exists independently of the G1 phase. The majority of cells begin to retract their primary cilia at the cell-cycle reentry (the G0/G1 transition) [21–23, 27–30]. Since Tucker et al. first reported the reciprocal relationship between ciliation and cell proliferation in cultured cells [27, 28], ciliary absorption (deciliation) has been well analyzed in cell culture [21, 29, 30]. To analyze the deciliation, cultured cells are typically starved of serum and then treated with serum or defined growth factors to induce deciliation. Recent studies have demonstrated that some manipulations can induce ciliogenesis in the presence of serum sufficiently high to allow cell proliferation [31–36]. These observations suggest a novel concept that cycling cells continuously suppress ciliogenesis. In this review, we describe this emerging concept, comparing with the phenomena of ciliary disassembly linked to cell-cycle reentry. We also discuss the negative impacts of primary cilia on cell-cycle progression.

### Cilia and cell cycle

In general, most cells begin to assemble primary cilia in response to cellular quiescence (which means the G0 phase) and destabilize them after cell-cycle reentry. Tucker et al. first described the relationship between deciliation and cell-cycle progression: the deciliation after cell-cycle reentry appears to complete prior to DNA replication [accompanied with centriolar (centrosomal) duplication] [27, 28]. Since then, several researchers have reported that primary cilia are completely disassembled prior to the S or M phases [21, 23, 29, 30].

However, some species reportedly retain their cilia during cell proliferation [30]. For example, many ciliated protozoans maintain their cortical cilia throughout cell division [37]. In the fruity fly (*Drosophila melanogaster*), spermatocytes undergo two meiotic divisions, keeping their cilia [38]. Therefore, the impact of primary cilia on cell-cycle progression has been a matter of debate.

On the other hand, recent studies have provided some hints about the relationship between primary cilia and cell cycle. Forced ciliation or deciliation can affect cell-cycle progression to the S or M phases [31–34, 39–43]. Since most ciliary regulators also exist outside cilia or centrosomes where they play distinct roles [23, 44], we should keep in mind that extra-ciliary or extra-centrosomal effects can be caused by each manipulation to induce (de)ciliation. However, several studies have clearly demonstrated that cell-cycle phenotypes by forced ciliation are reverted by simultaneous manipulations to destabilize cilia (ex. the co-impairment of IFT88, IFT20, or Talpid3), whereas only each destabilization treatment exerts minor effects on cell-cycle profile [31–33, 39, 40, 43]. These data have raised the possibility that primary cilia can function as negative regulators of cell cycle.

There are two major models, in which primary cilia negatively influence cell-cycle progression. One model is that ciliary length in quiescent cells may determine the G1 duration after cell-cycle reentry [30]. This model is supported by the following observations. Longer cilia in quiescent cells delay the progression to the S phase after cell-cycle reentry [39]. On the other hand, the loss of primary cilia in quiescent cell accelerates S-phase entry after serum stimulation [42]. The other is that the presence of cilia itself can function as a brake to cell-cycle progression to the S or M phases. Since centrosomes are relatively immobilized just beneath the apical membrane in ciliated cells, non-ciliated centrioles (centrosomes) may be required to serve as templates for centriole duplication during the S/G2 phase or to form spindle poles during mitosis in most cell types. This model accounts for the majority of published evidence regarding cell-cycle progression after growth factor stimulation [34, 39, 40, 43]. It is also applicable to the fact that forced ciliation in cycling cells arrests cell cycle [31–33] or reduces the proliferation rate [34].

### Ciliary resorption after cell-cycle reentry

In 1979, Tucker et al. reported that Balb/c or Swiss 3T3 fibroblastic cells assembled their primary cilia under cultivation at high cell density or with low serum [27]. They observed two waves of deciliation when these quiescent cells were stimulated with serum or defined growth factor [27, 28]. The first, initial deciliation occurred within 1–2 h, but cells were ciliated again by 6–8 h after serum stimulation. The second deciliation and final deciliation were detected at 12–24 h when cells replicated their DNA [27, 28]. In RPE1 cells [retinal pigment epithelial cells immortalized by human telomerase reverse transcriptase (hTERT)], the first and second waves are likely associated with the G0/G1 and G2/M transitions, respectively [45].

Several groups reported proteins implicated in the first wave of deciliation after serum stimulation [30]. Especially, the importance of mitotic kinases starts to emerge (Fig. 1). Since Aurora-A and PLK1 exhibit maximum activities in mitosis and the inhibition/depletion of either kinase results in several mitotic defects, they are basically categorized as mitotic kinases [46–50]. Using the green alga *Chlamydomonas reinhardtii*, Snell’s group first described that CALK (a protein kinase distantly related to mammalian Aurora-A) plays critical roles in the disassembly of motile flagella, structures evolutionally related to cilia in higher eukaryotes [51]. Using the mammalian cultured cells, Golemis et al. reported that Aurora-A participates in ciliary resorption after serum stimulation [45]. In this deciliation pathway, Aurora-A activation requires HEF1 (a protein which they previously identified as a novel Aurora-A-binding protein [52]; Fig. 1) [45]. Now, Pifo [53] and Ca^2+^/CaM [54, 55] are identified as additional Aurora-A activators in the ciliary resorption [23, 47]. Golemis’s group also identified HDAC6 as a downstream substrate for Aurora-A [45] (Fig. 1). Aurora-A-mediated phosphorylation stimulates the catalytic activity of HDAC6, resulting in axonemal tubulin deacetylation [45] (Fig. 1). This deacetylation is considered to destabilize axonemal microtubules (which means ciliary resorption), but the relationship between tubulin acetylation and microtubule stability is still being debated [23, 56, 57]. On the other hand, PLK1 is localized at the transition zone of cilia and participates in ciliary resorption after serum stimulation [58] (Fig. 1). PLK1 stabilizes HEF1 via non-canonical Wnt pathway, resulting in the activation of Aurora-A-HDAC6 deciliation pathway [59] (Fig. 1). PLK1 also phosphorylates KIF2A, which stimulates its microtubule-destabilizing activity. The elevation of KIF2A activity is required for the ciliary resorption after serum stimulation [60] (Fig. 1). Other proteins were also reported to participate in the first wave of deciliation after the serum stimulation [30]. These proteins include the components of cytoplasmic dynein (such as LC8 [39] and Tctex-1 [40]), NDE1 [39], Ndel1, LIS1 [32], CPAP [34], VDAC3, and MPS1 [35]: their function(s) are described in different chapters.

The following signaling pathways are reportedly involved in the second wave of ciliary resorption after the serum stimulation. PLK1 associates with HDAC6 and then activates it, a process required for the second wave of deciliation before mitosis [61] (Fig. 1). NEK2, a kinase involved in centrosome separation after centrosome duplication, [62] phosphorylates KIF24, which stimulates its microtubule-destabilizing activity [43] (Fig. 1). The inhibition of this signaling pathway delays the second wave, but not the first wave of deciliation [43]. Since HEF1 is transiently expressed at the G0/G1 and G2/M transitions in RPE1 cells [45], HEF1-Aurora-A complex may control both the waves of deciliation (Fig. 1).

### Ciliary resorption and cell-cycle progression

Given that the majority of ciliary proteins, including tubulins, are constantly turning over even after primary cilia remain constant in length, they are controlled by a dynamic equilibrium between assembly and disassembly (Fig. 1). This equilibration is maintained mainly by bidirectional transport system along the axoneme. Kinesin-2 family protein and IFT complex B (including IFT88 and IFT20) contribute to anterograde transport (from ciliary base to tip), whereas cytoplasmic dynein-2 and IFT complex A participate in retrograde transport (from ciliary tip to base) [15, 63]. In general, the loss-of-function of each anterograde transport protein (including IFT88 or IFT20) makes cilia shorter or absent, whereas that of each retrograde transport protein makes them swollen at the tip [15].

The studies of two dynein-related proteins first demonstrated that ciliary resorption after cell-cycle reentry affects subsequent cell-cycle progression [39–41]. Tsiokas et al. reported that NDE1, a protein modulating dynein activity [64, 65], negatively controls ciliary length via the interaction with a dynein light chain subunit LC8 [39]. In quiescent cells, NDE1 depletion lengthens cilia, whereas its overexpression renders them shorter and bulbous at the tip. Following the NDE1 depletion, cells develop longer cilia, accompanied by a delayed onset of DNA replication upon serum stimulation. This cell-cycle phenotype depends on the presence of cilia, because it is reverted by co-knockdown of IFT88 or IFT20, leading to forced ciliary absorption, although the depletion of IFT88 or IFT20 alone has a little impact on cell-cycle progression. The authors’ group also demonstrated that the timing of DNA replication after serum stimulation is delayed by other treatments that lengthen cilia, such as the induction of a constitutively active mutant of Rab8a and a brief treatment with an actin-depolymerizing reagent cytochalasin D [39].

Sung et al. reported that Tctex-1, a protein originally characterized as a light-chain subunit of cytoplasmic dynein [66, 67], is phosphorylated at Thr94 and then recruited to the transition zone on the cilia after the serum stimulation [40]. Tctex-1 knockdown or expression of a phospho-deficient mutant delays not only the first wave of ciliary absorption but also the timing of DNA replication after serum stimulation. Conversely, the replacement with its phospho-mimic mutant promotes both deciliation and progression to S phase after serum stimulation. This cell-cycle phenotype is observed in RPE1, 3T3, and MEF (mouse embryonic fibroblast) cells but not in HeLa (human cervical carcinoma) and COS7 (transformed, monkey kidney fibroblast) cells [40]. The former cell types can assemble primary cilia in response to cellular quiescence and additional cues, whereas the latter is categorized as non-ciliated cells. In addition, the cell-cycle phenotype by Tctex-1 depletion is relieved by two different treatments to promote ciliary disassembly [40].

Gopalakrishnan’s group have demonstrated the importance of CPAP (a protein known as a procentriole elongation factor [6, 14, 68, 69]) in both deciliation and cell-cycle progression after cell-cycle reentry [34]. CPAP mutation observed in Seckel syndrome [70] delays ciliary resorption processes after serum stimulation [34]. Since CPAP recruits NDE1, Aurora-A, and OFD1 to ciliary base likely through CPAP interaction with each molecule, CPAP may function as a scaffold for these deciliation factors [34] (Fig. 1). In addition, CPAP mutation reduces the percentage of cyclin-A-positive (likely S phase) and mitotic cells after cell-cycle reentry [34].

These observations raise the question of whether forced deciliation affects cell-cycle progression after cell-cycle reentry. The loss of cilia by CEP164 knockdown in quiescent cells accelerates the progression to S phase after the serum stimulation [42]. All these data support the idea that the first wave of deciliation after the cell-cycle reentry is required to start DNA replication, a phenomenon coupled with centriole duplication.

NEK2-KIF24 participates in the second but not the first wave of deciliation [43] (see the previous chapter). The ablation of NEK2 or KIF24 reduces the percentage of Ki-67-positive, proliferating cells [43]. Since this cell-cycle phenotype is relieved by the co-depletion of Talpid3 (a protein identified as a CP110-interacting protein and required for ciliogenesis [71]), the cell-cycle phenotype depends on the presence of cilia [43]. Therefore, the second wave of deciliation may be also required for subsequent cell-cycle progression (likely entry into mitosis).

### Suppression of ciliogenesis in proliferating cells

We describe recent findings concerning the inhibition of ciliogenesis in cycling cells. The experimental condition is quite different from the condition to analyze ciliary resorption after serum starvation. It is based on the serum concentrations sufficient to induce cell growth. We recently reported that Aurora-A knockdown induces ciliogenesis in RPE1 cells in the presence of serum [31]. This feature is phenocopied by the knockdown of trichoplein [31], a centriolar protein [72] originally identified as a keratin intermediate-filament-binding partner [73]. Trichoplein directly binds and activates Aurora-A in vitro and the two proteins are colocalized at the centrioles of proliferating cells, especially in the G1 phase [31]. Knockdown of either protein induces cell-cycle arrest at the G0 (or G1) phase [21, 31]. This cell-cycle arrest is reverted by treatments to promote ciliary disassembly [23, 31]. In HeLa cells (which are generally categorized as non-ciliated cells), trichoplein knockdown has a little impact on cell-cycle profile, whereas Aurora-A depletion mainly induces mitotic defects [31]. Our findings first provided the novel concept of ciliogenesis strictly inhibited in cycling cells. Aurora-A activation by trichoplein is of critical importance to suppress ciliogenesis in proliferating cells.

More recently, we found that the above features of trichoplein or Aurora-A knockdown are phenocopied by the depletion of Ndel1 (a well-known modulator of dynein activity [64, 65, 74]) in RPE1 cells [32]. This Ndel1 function might be independent of dynein activity [32]. Rather, Ndel1 might protect mother-centriole-associated trichoplein from the ubiquitin/proteasome-dependent degradation [32], a pathway mediated by Cul3-RING E3 ligase-KCTD17 complex (CRL3^KCTD17^) [33]. Thus, Ndel1 functions as an upstream regulator of the trichoplein-Aurora-A pathway to suppress ciliary assembly in cycling cells. Gopalakrishnan’s group reported that CPAP mutation not only induces ciliogenesis but also reduces cell proliferation rate under the growth condition [34]. Since CPAP is likely to be a scaffold protein for Aurora-A [34], CPAP may also function upstream of the trichoplein-Aurora-A pathway.

The other pathways were reported to participate in ciliary suppression in growing cells. The mitochondrial porin VDAC3 and MPS1 (a kinase functioning at centrosomes and kinetochores [75]) suppress ciliary assembly in cycling RPE1 cells [35], but the underlying mechanisms remain largely unknown. The overexpression of miRNA-129-3p, a microRNA conserved in vertebrates, also induces ciliogenesis in RPE1, ARPE19, and IMCD3 cells under the growth condition, whereas it fails to cause severe cell cycle arrest in RPE1 cells [36]. This microRNA reduces the expression of CP110 (a capping protein at the distal end of centrioles [16, 17]) and multiple actin regulator gene products [36].

Accumulating evidence has proposed a model stating that cycling cells suppress ciliogenesis. However, there exists a counterargument that the appearance of cilia in growing cells may be due to a failure to absorb cilia existing in G1 phase. Indeed, it is difficult to completely rule out the possibility that every single cell generates a primary cilium after mitosis and then destabilizes it in accordance with cell-cycle progression, partly because minor fraction (~5–15 %) of RPE1 cells possess primary cilia under the cultivation with enough serum [31–36]. Reportedly, ciliary resorption after cell-cycle reentry is affected by several suppressors of ciliogenesis in proliferating cells, such as Ndel1 [32], VDAC3, MPS1 [35], or CPAP [34]. However, using deciliation assays, it is difficult to distinguish whether ciliary resorption is delayed or once-deciliated cells regenerate primary cilia after the G0/G1 transition. In addition, not all molecules for ciliary resorption are involved in the suppression of ciliogenesis in proliferating cells. For example, ciliary resorption is delayed by the inhibition of components of cytoplasmic dynein (including Tctex-1 [40]), NDE1 [39], Ndel1, and LIS1 [32], whereas only Ndel1 or LIS1 depletion induces ciliogenesis under the cultivation with serum [32]. Thus, it is more conceivable that there are at least two categories of machineries to negatively regulate ciliogenesis: one is to destabilize existing primary cilia (after cell-cycle reentry) and the other is to suppress primary cilia assembly (during cell proliferation). We consider a model stating that the resorption of existing primary cilia may require more driving forces than the suppression of ciliary assembly. In other words, many more negative regulators may work for the disassembly of existing primary cilia than the maintenance of deciliated mother centriole. This model is appealing in the light of published evidence that more molecules are identified for ciliary resorption.

### The behavior of negative regulators in ciliogenesis

A recent genome-wide RNAi screening by Lee et al. indicated that ciliation or deciliation coupled with cell cycle requires a lot of proteins involved in mRNA processing and ubiquitin–proteasome system (UPS) [76]. This study may suggest that (de)ciliation is regulated by protein synthesis and destruction coordinated with cell cycle [76]. Several excellent reviews have described the importance of timely protein synthesis or destruction in ciliogenesis or deciliation, respectively [14–20]. As described in the previous chapters, we mainly discussed the synthesis of several proteins implicated in deciliation coupled with cell-cycle reentry. In this chapter, we mainly focus on protein destruction implicated in ciliation coupled with cell-cycle exit.

Upon cell-cycle exit, NDE1 is degraded by an SCF^Fbw7^-dependent UPS. NDE1 recognition by SCF^Fbw7^ requires NDE1 phosphorylation by CDK5, a kinase activated during quiescent state [77]. However, NDE1 depletion makes cilia longer even in quiescent cells [39]. Therefore, the protein level of NDE1 is lower at the G0 phase than at the G1 phase but NDE1 negatively controls ciliary length even during quiescent state (Fig. 1) [39]. On the other hand, the protein level of Tctex-1 does not dramatically change between quiescent and proliferation states. Whereas Tctex-1 may also have negative impacts on ciliary length in quiescent cells [78], the change in Tctex-1 localization is critical for ciliary resorption after cell-cycle reentry [40]. Upon cell-cycle reentry, Tctex-1 is phosphorylated at Thr94. This phosphorylation is of critical importance in both Tctex-1 recruitment to ciliary transition zone and ciliary resorption (Fig. 1) [40]. Thus, the two dynein-related proteins may negatively control ciliary length even during quiescent state when the length becomes constant, but their activities to disassemble cilia are elevated in response to growth stimulation. LIS1, a protein to modulate dynein activity [79, 80], and dynein complexes not only limit ciliary length in quiescent cells but also regulate ciliary resorption after cell-cycle reentry [32]. However, whether the level or activity of these proteins is changed at the G0/G1 transition remains largely unknown.

Reportedly, trichoplein is removed specifically from the mother-centriole/basal body at the transition from G1 to G0 phase. Since exogenous induction of trichoplein inhibits ciliogenesis in quiescent cells, this removal is required for ciliogenesis in response to serum depletion [31]. We also found that trichoplein is degraded through its polyubiquitination by CRL3^KCTD17^ [33]. However, the activity of this ubiquitin ligase is unlikely to dramatically change between proliferation and quiescent states. So, there exits mechanism(s) by which trichoplein destruction preferably occurs during the G0 phase. Interestingly, Ndel1 is degraded more rapidly than trichoplein in response to serum depletion. Exogenous Ndel1 expression suppresses trichoplein degradation and ciliogenesis in response to serum depletion. Thus, Ndel1 degradation is required for CRL3^KCTD17^-mediated trichoplein polyubiquitination upon cell-cycle exit. Ndel1 is also destructed by UPS (other than CRL3^KCTD17^-mediated pathway), which is estimated to be more active in the G0 phase [32]. However, the responsible E3 ligase has not identified yet.

### Cell cycle and centrosomal morphology: possible existence of structural checkpoint

**Fig. 2:**
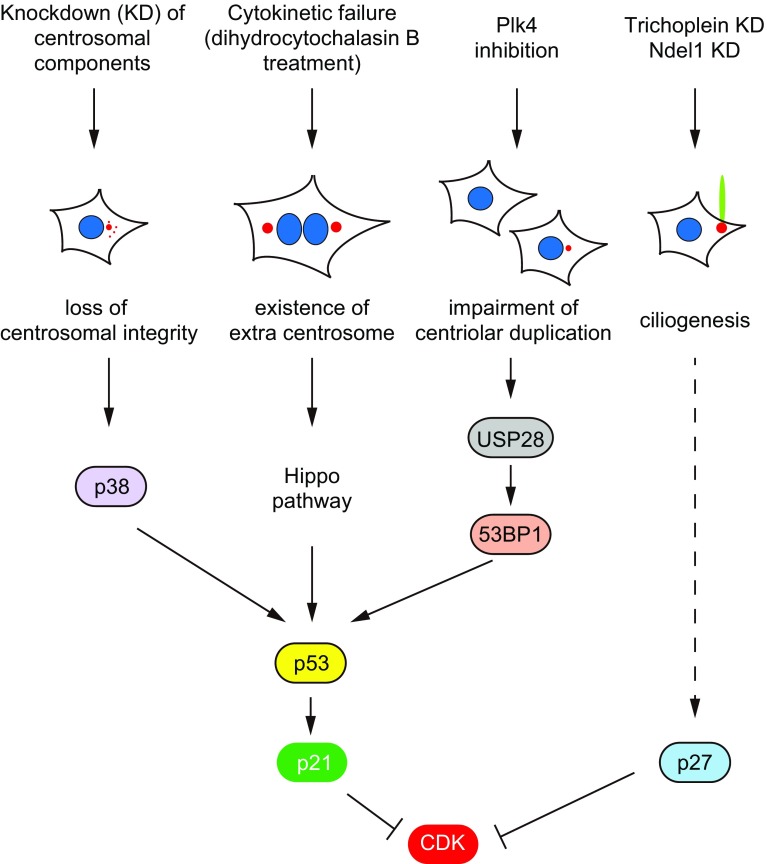
Possible cell-cycle checkpoint pathways in centrosomal (centriolar) integrity or morphology. Loss of centrosomal integrity by knockdown of centrosomal components activates p38–p53–p21 pathway. The existence of extra centrosome induced by cytokinetic failure activates Hippo signaling pathway, resulting in p53 stabilization. Impairment of centriolar duplication by PLK4 inhibition activates USP28–53BP1–p53–p21 pathway. Ciliogenesis induced by the knockdown of trichoplein or Ndel1 in proliferating cells results in the elevation of p27. P21 or p27 may suppress CDK activities, resulting in cell-cycle arrest

Whether the status of centrosome (or centrioles) affects cell cycle remains a matter of debate, but recent studies have pointed out the importance of p53-dependent checkpoint pathway in centrosomal (centriolar) integrity (Fig. 2). Doxsey et al. reported that the loss of 14 out of 15 centrosomal proteins activates p38–p53–p21 pathway [81] (Fig. 2). Since p21 is one of cyclin-dependent kinase (CDK) inhibitors (CKIs) [82, 83], the elevation of p21 protein level results in cell-cycle arrest at the G1/S transition [81] (Fig. 2). Pellman’s group also reported that extra centrosome formation due to cytokinetic failure activates the Hippo signaling pathway, resulting in G1-arrest due to p53 stabilization [84] (Fig. 2). More recently, Holland’s and Oegema’s groups simultaneously reported that USP28–53BP1–p53–p21 checkpoint pathway is activated by the impairment of centriolar (centrosomal) duplication due to PLK4 inhibition [85, 86] (Fig. 2). All these studies have proposed a model that cells equip a p53-dependent surveillance mechanism for centrosomal (centriolar) integrity, whereas several signaling pathways may coexist upstream of p53.

On the other hand, less is known about how cells detect their (de)ciliation status and then adjust S- or M-phase entry. However, recent studies provide some clues. As described in the chapter “Suppression of ciliogenesis in proliferating cells”, Aurora-A activation by trichoplein is required for the suppression of primary cilia, which enables cells to proliferate [31]. Since p53 is phosphorylated and then inactivated by Aurora-A [87, 88], it is possible that ciliated cells may also activate p53–p21 axis. However, this possibility is less, likely because trichoplein knockdown appears to rather decrease the protein level of p53 or p21 [21]. Instead, trichoplein depletion results in the elevation of p27 [21], one of CKIs (Fig. 2). P27 is known to increase during quiescent state but decrease during proliferation state [82, 83]. Since CDK activities are also critical for the G0/G1 transition through the phosphorylation of pRb (the product of the *retinoblastoma* tumor suppressor gene) [89–91], p27 may suppress CDK activities in ciliated cells (Fig. 2). Since the level of pRb phosphorylation is significantly reduced by Tetex-1 depletion which delays ciliary resorption after cell-cycle reentry [40], CDK activities may be also suppressed when ciliary resorption is delayed. These observations have raised the possibility that cells with primary cilia exert a mechanism similar to cell-cycle checkpoint machinery at the G0 phase.

### Ciliopathy and cancer

Recent studies have highlighted a possible role of primary cilia for delay in cell-cycle progression or cell-cycle arrest. This negative impact of primary cilia has raised a model, in which the absence of primary cilia leads to the growth advantage. Newborn mice with reduced expression of Ndel1 exhibit both an increase in primary cilia and the reduced proliferation rate in kidney tissues [32]. Patients with PKD generate benign kidney cysts, which are likely associated with cell overgrowth phenotype [6, 92, 93]. Patients with Birt–Hogg–Dubé syndrome [94] and Von Hippel–Lindau (VHL) syndrome [95] not only exhibit some clinical features of ciliopathies but also predispose to renal cancers [93]. However, except for these two syndromes, cancer incidence is not increased in human ciliopathies [93].

It is not clear why human ciliopathies are not generally predisposed to cancer, but one possible explanation is that primary cilia appear to have diverse effects on cell proliferation. For example, primary cilia are required for cell proliferation in neuroepithelial cells. It is generally considered that primary cilia are essential to receive extracellular growth signals (such as a Hedgehog morphogen) in these cells [96–100]. Interestingly, Sung’s group has demonstrated that primary cilia are disassembled after receiving growth signals and this ciliary resorption may be required for subsequent cell-cycle progression in neuroepithelial cells [101]. In addition, the frequency of ciliated cells is generally reduced in the majority of tumor tissues/cell lines, but some types of cancer cells clearly propagate in a primary cilia-dependent manner [6, 99, 100, 102], like neuroepithelial cells. This complexity may affect the pathological appearances of each ciliopathy.

## Conclusion and perspectives

The purpose of this review is to introduce the emerging concept that cycling cells continuously suppress ciliogenesis, comparing with the mechanisms underlying ciliary resorption after cell-cycle reentry. We have also highlighted the reciprocal relationship between primary cilia and cell-cycle progression. However, the impact of primary cilia on cell proliferation is not so simple. Primary cilia can act as the negative regulators of cell-cycle progression, whereas primary cilia are also required for cell proliferation to receive extracellular growth signals. More investigations about these complex roles will lead to a better understanding not only of ciliopathies but also of cancers.
